# Comparative Study of Lipid- and Polymer-Supported Membranes Obtained by Vesicle Fusion

**DOI:** 10.1021/acs.langmuir.2c00266

**Published:** 2022-04-26

**Authors:** Rachel J. Goodband, Colin D. Bain, Margarita Staykova

**Affiliations:** † Department of Physics, Durham University, Durham DH1 3LE, U.K.; ‡ Department of Chemistry, Durham University, Durham DH1 3LE, U.K.

## Abstract

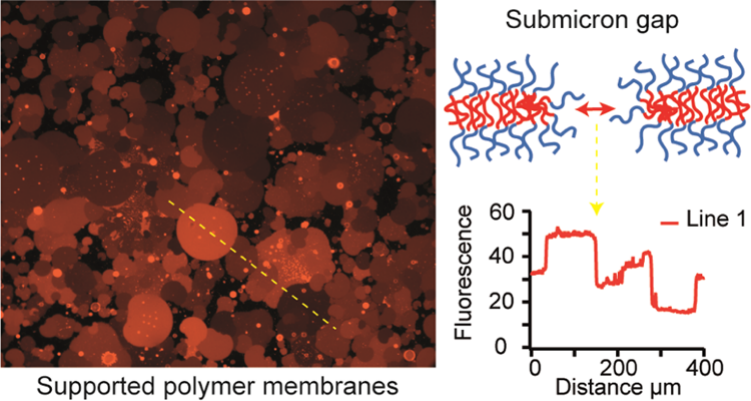

We compare the fusion of giant lipid and block-copolymer vesicles on glass and poly(dimethylsiloxane) substrates. Both types of vesicles are similar in their ability to fuse to hydrophilic substrates and form patches with distinct heart or circular shapes. We use epifluorescence/confocal microscopy and atomic force microscopy on membrane patches to (i) characterize bilayer fluidity and patch-edge stability and (ii) follow the intermediate stages in the formation of continuous supported bilayers. Polymer membranes show much lower membrane fluidity and, unlike lipids, an inability of adjacent patches to fuse spontaneously into continuous membranes. We ascribe this effect to hydration repulsion forces acting between the patch edges, which can be diminished by increasing the sample temperature. We show that large areas of supported polymer membranes can be created by fusing giant vesicles on glass or poly(dimethylsiloxane) substrates and annealing their edges.

## Introduction

Block copolymers with one hydrophilic and one hydrophobic segment self-assemble in water into micelles, vesicles, or bilayers. In the last 20 years, block-copolymer bilayers have been extensively explored as membrane materials^1,2^ for applications such as encapsulation (building nanoreactors,^3^ drug delivery^4^) and surface coatings (water separation,^5^ biosensors^6^). Like lipids, block copolymers can undergo phase transitions, membrane fusion, and fission.^7^ However, lipid and polymer membranes have distinct physical differences stemming from the size and structure of their molecular building blocks. Block copolymers used in membranes are often larger than lipids, sometimes with side branches, enabling them to assemble into thicker, more elastic (higher bending rigidity and stretching elasticity) membranes, with low permeability (even to small molecules) and reduced lateral fluidity.^8^ In addition, the structure and chemical composition of the polymers can be easily manipulated, allowing fine control over the properties and the morphology of the membranes they form.

The formation of solid-supported polymeric bilayer membranes remains a challenge,^9,10^ even though various techniques such as vesicle^11−14^ and micelle deposition^15^ or Langmuir dipping^16,17^ have been tested. Functionalized polymer end groups,^11,14,16,17^ surface modifications,^18^ or electrostatic attraction^13^ have been used to bind the polymer to the substrate. As a result, the lower membrane leaflet often becomes immobilized (or partially immobilized) on the substrate,^13,16,17^ generating an overall low membrane fluidity, compared to its otherwise fluid vesicle form.^19−21^ There are a small number of cases where poly(ethylene oxide)-block-polybutadiene (PEO14-PDB22) polymer bilayer membranes, which are the subject of this study, have been formed successfully without surface or polymer modification, but these membranes are not fluid.^12,15^ For example, using fluorescence recovery after photobleaching (FRAP), Gettel et al.^12^ showed that pure PEO14-PBD22 bilayers, formed by fusing small unilamellar vesicles (SUVs) on hydrophilic glass, showed no fluorescence recovery over 90 min, in contrast to mixed polymer–lipid samples, which recovered within minutes. Similarly, Goertz et al.^15^ found no fluidity in PEO30-PBD22 bilayers on hydrophilic glass but some, albeit low, mobility in monolayers on hydrophobic glass substrates. The lack of mobility in the hydrophilic systems was attributed to the strong coupling between the PEO groups and the substrate, possibly due to hydrogen bonds.^15^

In this work, we compared the formation and properties of supported membrane patches of PEO14-PBD22 and phospholipids.^22^ We characterized the fluidity and integrity of the patch membrane and the properties of its edges. We used the knowledge learned from patches to determine the conditions for forming continuous polymer membranes. We found that polymer GUVs fused readily to hydrophilic substrates, forming circular or heart-shaped patches as previously observed with lipids.^23^ In contrast to earlier reports,^12,15^ PEO14-PBD22 membranes retained fluidity when coupled to a substrate^12,24^ but were less fluid than lipid patches. In addition, we revealed significant differences in the edge stability and behavior of lipid and polymer patches, providing insights into why the formation of continuous supported membranes via polymer vesicle fusion cannot proceed spontaneously. Furthermore, we demonstrated that polymeric membranes can be successfully formed on poly(dimethylsiloxane) (PDMS) substrates, which opens up the possibility for future mechanical studies and applications.^25^ PDMS is a silicone elastomer, increasingly used for biomedical applications, wearable technologies,^26,27^ and for mechanical studies of cells and membranes. PDMS substrates can either be subject to tensile forces^25,27−30^ or molded in surface topologies of high precision and curvature,^31−34^ which significantly expands the application and studies of supported membranes.

## Materials and Methods

### Materials

Sucrose, glucose, sodium chloride (NaCl), Trizma base (Tris), calcium chloride (CaCl_2_), hydrochloric acid, and chloroform were all purchased from Sigma-Aldrich and used as received. PDMS devices were fabricated using Sylgard 184 Silicon Elastomer Kit. 1,2-Dipalmitoyl-*sn*-glycero-3-phosphoethanolamine-*N*-(lissamine rhodamine B sulfonyl) (ammonium salt) (Rh-DPPE) (*M*_w_ = 1301 g/mol) and 1,2-dioleoyl-*sn*-glycero-3-phosphocholine (DOPC) (*M*_w_ = 734 g/mol) were purchased from Avanti Polar Lipids. InvitrogenTM, 1,1′-dioctadecyl-3,3,3′,3′-tetramethylindodicarbocyanine perchlorate (DilD) (*M*_w_ = 1052 g/mol) was obtained from ThermoFisher Scientific and Naphtho[2,3-*a*]pyrene (Naphth) (*M*_w_ = 302.37 g/mol) from Santa Cruz Biotechnology (Heidelberg, Germany). Poly(1,2-butadiene)-*b*-poly(ethylene oxide) (PEO14-PBD22) (*M*_w_ ∼ 1960 g/mol) was obtained from Polymer Source. All water used was ultrapure (Milli-Q) water.

### Methods

#### Preparation of Vesicles

We prepared lipid and polymer GUVs from the stock solutions of PEO14-PBD22 or DOPC (4 mg/mL) in chloroform containing 1 mol % of the respective fluorophore, using both the electroformation method^35^ and gentle hydration on cellulose paper.^36^ For electroformation, we dried 10 μL of the polymer or lipid stock solution onto ITO glasses of the electroformation chamber and rehydrated it in 300 mM sucrose to a final lipid/polymer concentration of 0.2 mg/mL. We applied a 3 Hz AC electric field of 0.5pp V/mm between the ITO plates for 16 h at 60 °C.

GUVs were also formed by gentle hydration on cellulose paper for comparison. We dried 10 μL of the polymer stock solution onto Whatmann size 1 paper, dessicated to remove residual chloroform, and rehydrated with 300 mM sucrose, as described elsewhere.^36^

To prepare SUVs, we dried the equivalent of 1 mg (dry weight) polymer/flurophore solution onto the walls of a glass vial, dessicated to remove residual chloroform, and then resuspended it in sucrose and tip sonicated (Cole Parmer ultrasonic processor CFX130, 130 W, 25%) it until we obtained a clear solution of suspended SUVS.

#### Vesicle Fusion and Formation of Supported Patches

To prepare PDMS substrates, we spin-coated the PDMS polymer melt (polymer/crosslinker 10:1) onto glass slides and cross-linked it at 60 °C overnight. This resulted in a thin (around 50 μm thick), transparent layer of PDMS on a glass substrate that is naturally hydrophobic but which could be made hydrophilic via plasma treatment (Tantec VacuLAB, 300 W, 1 mbar, air) for 20 s. Glass substrates were cleaned in IPA and milliQ water, plasma-oxidized (BIO RAD E2000, 40 W, 1 mbar, air, 10 min), and heated for 1 h at 200 °C. We verified that both methods for plasma treatment produced identical effects and were not the cause for the observed differences between PDMS and glass. Prepared in this way, our plasma-oxidized PDMS substrates exhibit uniform hydrophilic surface properties,^37^ which are retained within the time scales of our experiments (maximum 3 h).^38^

For the imaging, we prepared a chamber by attaching a PDMS spacer to the prepared glass or PDMS substrates. We filled the chamber with fusion buffer and added the desired amount of GUV suspension, depending on the density of patches we required. We compared two fusion buffers: (i) an iso-osmotic buffer (150 mM NaCl, 2 mM CaCl_2_, and 10 mM Tris) that matches the osmolarity of the vesicle interior and (ii) a hypo-osmotic fusion buffer (75 mM NaCl, 1 mM CaCl, and 5 mM tris), which subjected the vesicles to hypo-osmotic shock of 150 mM. The GUV suspension was left for 3 min to fuse to the substrate and then rinsed using the same buffer to remove excess material prior to imaging.

#### Imaging and Analysis

Laser scanning microscopes (Leica SP5 and Zeiss LSM 900) were used to collect high-resolution fluorescent images of the membranes and for FRAP measurements on polymer bilayers. A JPK NanoWizard atomic force microscope (AFM) was used to take height maps of the patches in quantitative imaging mode and in contact. Bruker Scan Assist fluid (nominal stiffness 0.7 N/m) and Bruker SNL-10 tips (nominal stiffness 0.24 N/m) were used to collect AFM images.

The diffusion coefficient, *D*, and the fraction of immobile fluorophores in the supported bilayer sample were quantified by FRAP. We photobleached a 6 μm diameter area in the middle of individual membrane patches and tracked the recovery over time. Several different patches on each of 3–4 samples were studied. To correct for photobleaching, each recovery curve was normalized by the fluorescence of a neighboring non-photobleached patch (Figure S2). To obtain the diffusion coefficient from photorecovery, we used SimFRAP,^39^ a FIJI-based plugin that fits a simulated diffusion (a 2D random walk) to experimental FRAP data sets. Ten runs of the simulation were made and averaged on each patch. All errors are quoted as the standard error in the mean.^40^ The patch-to-patch variation is much larger than the sample-to-sample variation and hence forms the dominant error on patch fluidity. The immobile fraction of fluorophores was obtained from the ratio of the initial (prebleach) and final fluorescent intensities of the photobleached spot. We also used FRAP to determine the patch-to-patch connectivity (Patch-to-Patch Variation in Fluorescence Intensity section) for which we photobleached much larger areas of 40 μm × 40 μm and recorded the time-dependent fluorescent recovery. To calculate the statistical significance of pairwise comparisons between independent samples with the unequal variance, we used Welch’s *t*-test with a significance level of 0.05.

## Results and Discussion

### Vesicle Fusion and Formation of Supported Polymer Patches

We first explored the optimal conditions for fusing polymeric GUVs (PGUV) on glass and PDMS and compared them to the fusion of lipid GUVs (LGUV). The fusion of lipid vesicles has been explored in detail for a range of substrates.^22,23,41−43^ We find that while the fusion of PGUVs proceeds in a similar fashion, there are some important differences. LGUVs fused instantaneously on hydrophilic glass and PDMS substrates using iso-osmotic buffer. In comparison, PGUVs fused quicker and produced patches with less excess membrane in hypo- than in iso-osmotic conditions. Most of the experiments reported here present membrane polymer patches formed in hypo-osmotic buffer, except where isotonic buffer is specified in the text. Furthermore, PGUVs fused readily on plasma-treated PDMS substrates at room temperature but not on plasma-oxidized glass, which had to be baked at 200 °C for 1 h to allow fusion. This treatment increased the contact angle of water on the substrate from 0° (fully wetting) to 31° (data not shown), possibly due to dihydroxylation of silanol groups, leaving fewer silanol groups on the substrate to form hydrogen bonds to water. Weakening of the hydration repulsion forces on baked glass has shown to favor fusion.^44^ The need for the baking step for the fusion of PGUVs, but not LGUVs, on glass, and the stronger hydration of the larger PEO headgroups suggest that hydration repulsion forces play a larger role in PGUV than LGUV fusion, and in fusion on glass than on PDMS.

**Figure 1:**
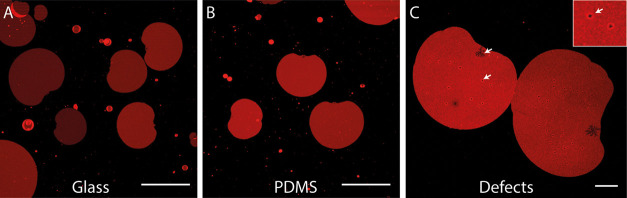
Confocal microscope images of PEO14-PBD22 patches in hypo-osmotic buffer deposited on (A) glass and (B) PDMS substrates; (C) defects observed in polymer membranes include fusion pores (fractal pattern, white arrow) or blebs (inset). Scale bars are 25 μm.

Polymer GUVs appear to fuse onto glass and PDMS substrates via a single-burst event, in contrast to lipid GUVs, which often fuse in a cascade fashion^43^ (Figure S1, Supporting Videos S1 andS2). This results in large polymer patches of distinct heart or circular shapes, with a low density of remaining daughter vesicles or other visible defects (Figure 1). Occasionally, pores appeared at the point of first contact between the PGUV and the substrate, as has been demonstrated for lipid vesicle fusion.^23,41^ However, the fusion pores in the polymer membrane showed fingering shapes (Figure 1C) in contrast to the round lipid pores. More frequent was the observation of bleblike protrusions (Figure 1C inset), especially after PGUV fusion in hypo-osmotic conditions, suggesting an origin related to osmosis. These defects were not present in every membrane patch. Vigorous rinsing/washing of the membranes created new defects and grew existing defects in the membrane.

### Fluidity of Supported Bilayers

**Figure 2:**
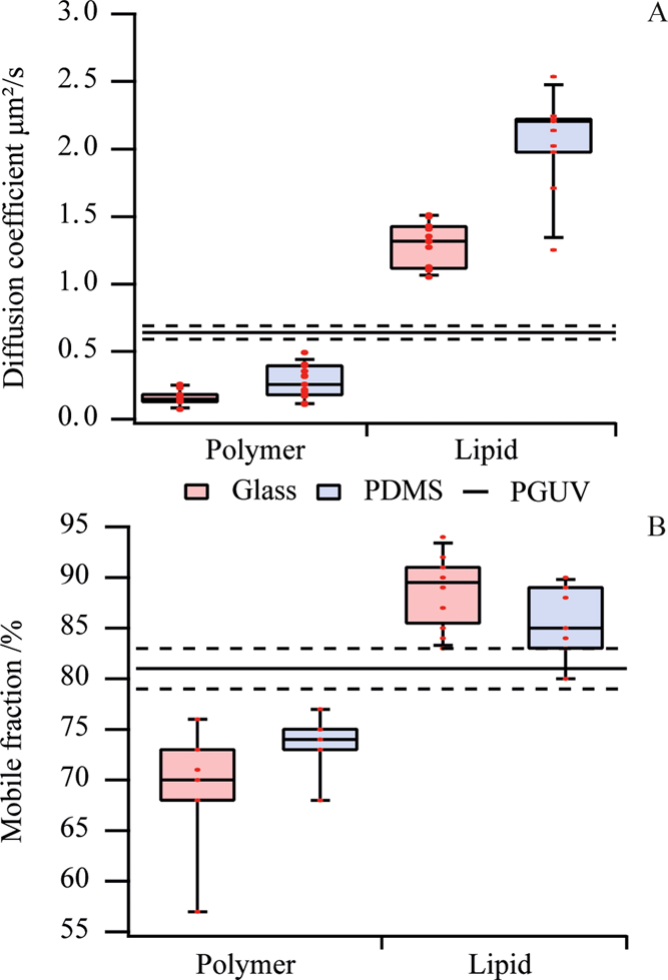
Comparison of (A) the diffusion coefficient and (B) the mobile fraction of polymer and lipid membranes deposited in hypo-osmotic buffer onto either (i) BKGL—plasma-treated glass, baked for 1 h at 200 °C (red box and whiskers), or (ii) on plasma-treated PDMS (blue box and whiskers). In both plots, the solid line represents measurements on the unsupported PGUV membrane and the dashed line the standard error. For polymers, *D*_PDMS_ = 0.28 ± 0.03 μm^2^/s, *D*_BKGL_ = 0.16 ± 0.01 μm^2^/s, and *D*_PGUV_ = 0.64 ± 0.06 μm^2^/s. For lipids, *D*_PDMS_ = 2.1 ± 0.1 μm^2^/s and *D*_BKGL_ = 1.29 ± 0.05 μm^2^/s. The mobile fraction for polymers is *I*_PDMS_ = 74 ± 1%, *I*_BKGL_ = 69 ± 2%, and *I*_PGUV_ = 81 ± 2%. The mobile fraction for lipids is *I*_PDMS_ = 85 ± 1% and *I*_BKGL_ = 89 ± 1%.

To check the fluidity of the supported PEO14-PBD22 membranes, we measured the diffusion of Rh-DPPE in the membranes using FRAP. Rh-DPPE is a headgroup-labeled lipid that has a similar molecular weight to PEO14-PBD22 (1301 and 1960 g/mol, respectively). Hence, even though we are not directly measuring the diffusion of polymers, the FRAP results give us a good estimate of the fluidity of polymer membranes and can be used for comparative studies. The diffusion coefficient, *D*, we obtain for lipid bilayers on glass and PDMS substrates is in close agreement with previous measurements in the literature (Figure 2A).^45,46^

In comparison, the diffusion coefficient of Rh-DPPE in supported PEO14-PBD22 membranes was 7–8 times smaller. Unsupported polymer membranes, as measured in PGUVs, showed a higher *D* but still lower than in supported lipid bilayers (Figure 2A), suggesting that interactions with the substrate account for only 2–4 times reduction in mobility. Overall, the diffusion coefficients of supported and unsupported PEO14-PBD22 membranes are in the range of lipid membranes in the gel phase^47^ (10^–2^–10^–1^ μm^2^/s).

Our measurements additionally reveal a dependence of *D* on the supporting substrate: diffusion was significantly lower on glass compared to PDMS, for both lipid and polymer membranes. This could be attributed to different substrate roughness and hydrophilicity, as discussed elsewhere.^48^

Using FRAP, we also compared the fraction of immobile fluorophores in the photobleached area (Figure S2), as a measure of bilayer inhomogeneities caused by the membrane constituents or their interaction with the substrate. Despite the large variation in the measurements, supported polymer bilayers displayed a consistently smaller mobile fraction (10–20% lower) than supported lipid membranes (Figure 2B), with unsupported polymer vesicle membranes having a value in-between.

### Patch-to-Patch Variation in Fluorescence Intensity

**Figure 3:**
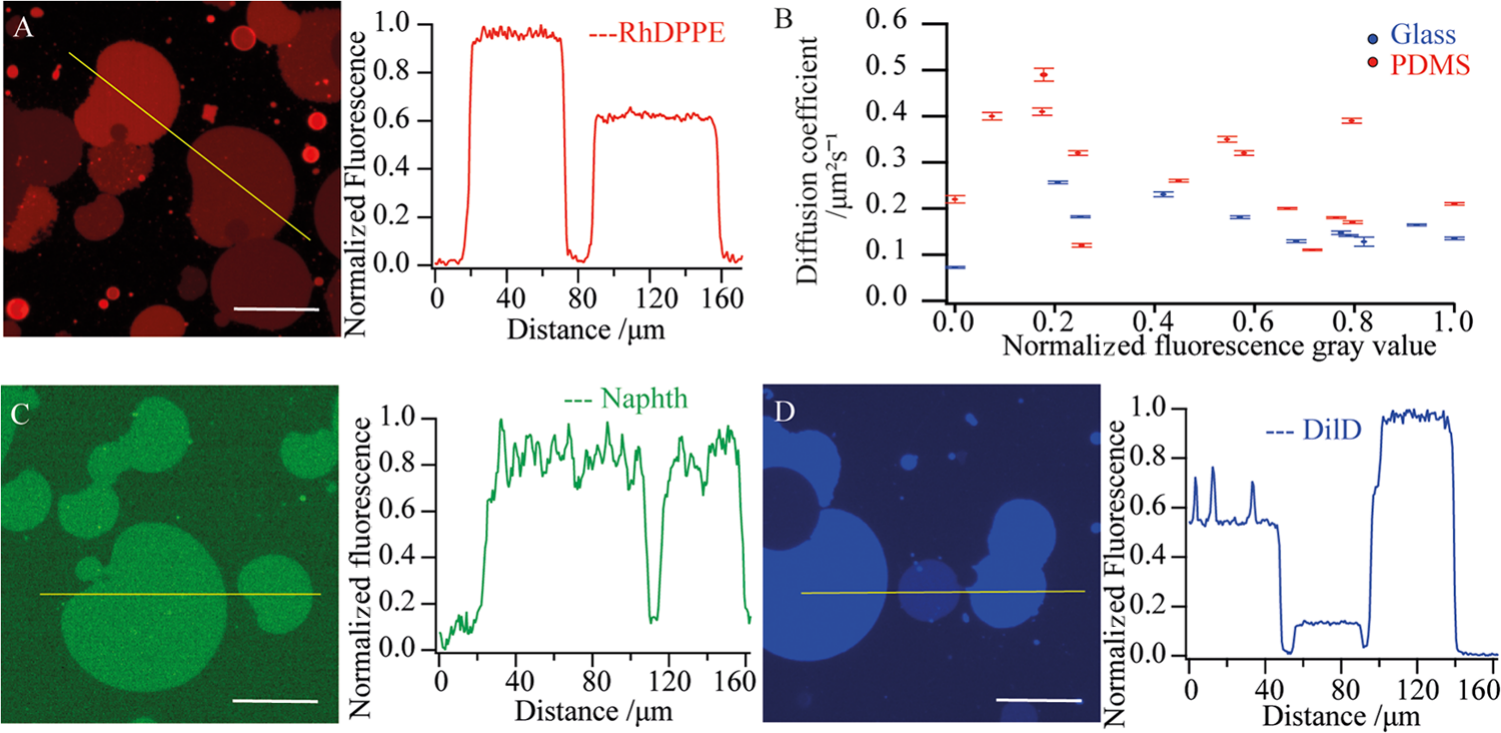
Fluorescence images of patches with (A) Rh-DPPE, (C) naphthopyrene, and (D) DilD fluorophores on PDMS substrates. The plots show the fluorescent intensity line profile through several patches following the yellow line in the respective fluorescent images. Scale bars are 50 μm. (B) Plot of patch intensity vs diffusion coefficient for polymer patches with the Rh-DPPE label on glass (blue) and PDMS (red) substrates. The error bars show the standard error.

A striking feature of the fluorescence images of polymer membrane patches was the intensity variation across patches—even ones that appeared to touch each other—in contrast to lipid patches, which have uniform intensity within a sample (Figures 3A and S1). This variation, by a factor of up to 3, was observed on both glass and PDMS and prompted further investigation.

First, we explored whether the differences in fluorescence intensity were caused by the formation of multilamellar membrane patches following PGUV fusion. None of our measurements supported this hypothesis. First, the patches showed a continuum of fluorescent intensities and not discrete multiples of the intensity from a single bilayer (Figure 3A). Second, the diffusion coefficient, which one would expect to be different in single or multiple bilayers, was not correlated to the fluorescence intensity of the patches (Figure 3B). Finally, the mean height of the polymeric membranes assessed by AFM was found to be 10 nm (Figure S3), regardless of the fluorescent intensity of the patch. The fact that we were able to observe similar variation between the fluorescence intensities of PGUVs before fusion (Figure S4) indicated that their origin was not related to the fusion process nor to interactions of the fluorophore with the substrate.^49^ We confirmed that the difference was not due to the method of vesicle formation, as both AC-electroformation^35^ and vesicle swelling on cellulose paper^36^ produced similar intensity variation between vesicles (Figure S4).

We then compared Rh-DPPE-labeled polymer patches with ones labeled by DilD and naphthopyrene. DilD has a similar molecular weight as the headgroup-labeled lipid Rh-DPPE but due to its hydrophobic nature localizes in the hydrophobic membrane core. Naphthopyrene is a small hydrophobic molecule with 3 times smaller molecular weight than the Rh-DPPE and DilD. We observed fluorescence variations only in patches labeled with Rh-DPPE and DilD but not with naphthopyrene (Figure 3). One possible explanation is that the larger fluorophores became unevenly distributed during the vesicle electroformation process due to their slow diffusion in the viscous polymer bilayer. This argument was partly supported by our FRAP measurements, which show the diffusion coefficients of Rh-DPPE and DilD were on average smaller than that of naphthopyrene (Figure S5). However, the spread of the measurements with naphthopyrene was large (due to its weak fluorescence and rapid photobleaching), and the difference with Rh-DPPE was not statistically significant (Figure S5). An alternative explanation is the limited solubility of amphiphilic Rh-DPPE and DilD molecules in the anhydrous block-copolymer film. The formation of aggregates and/or crystallites of DPPE or DilD would lead to an uneven distribution of fluorophores in the GUVs during formation. These inhomogeneities would equilibrate with time in the reconstituted vesicles giving the uniform fluorescence intensities, which we observe in GUVs. In contrast, naphthopyrene is hydrophobic and can disperse evenly in the hydrophobic regions of the cast block-copolymer films.

### Boundaries between Polymer Patches

**Figure 4:**
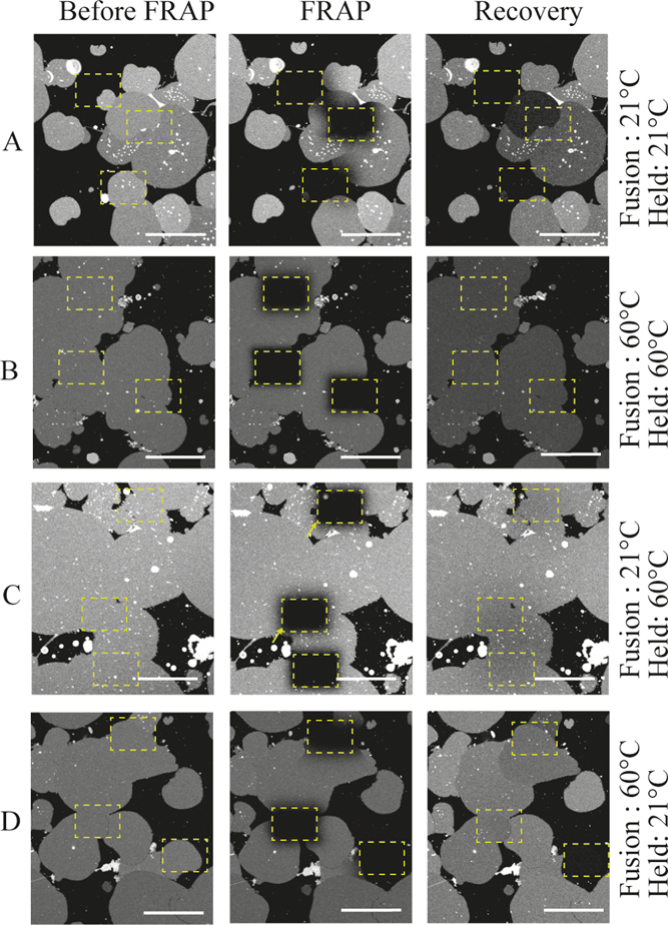
Fluorescence images of polymer patches (A) fused and incubated at 21 °C, (B) fused and incubated at 60 °C, (C) fused and incubated at 21 and 60 °C, respectively, and (D) fused and incubated at 60 and 21 °C, respectively, before, during, and after FRAP. The area of the photobleached region is approximately 40 μm × 40 μm and indicated by a yellow dashed rectangle in the images. The scale bars of images are 50 μm.

Figures 1 and 3 show that the fluorescence variations across patches did not equilibrate even when the patches were in contact. FRAP was further used to quantify the ability of the fluorophores to diffuse between adjacent patches (Figure 4). Patches fused and incubated at room temperature (21 °C) showed fluorescence variations which increased even further when a region of a patch was photobleached (Figure 4A). Samples fused and incubated at 60 °C showed no fluorescence intensity difference prior to photobleaching and recovered their fluorescence homogeneity after photobleaching (Figure 4B). To assess the relative importance of fusion and incubation in permitting diffusion between patches, we investigated two additional samples. Samples fused at room temperature and incubated at 60 °C for 2 h showed no patch variations before and after photobleaching (Figure 4C). However, samples fused (a process that takes seconds) at 60 °C and immediately cooled to room temperature showed variations in patch fluorescence intensity that did not change with time and which increased following partial or full photobleaching of patches (Figure 4D). Hence, the annealing of the edges between adjacent polymer patches was not fully achieved during fusion, even at 60 °C, and required a prolonged incubation at a high temperature.

We explored the morphology of the membrane edges at higher resolution by AFM imaging.

**Figure 5:**
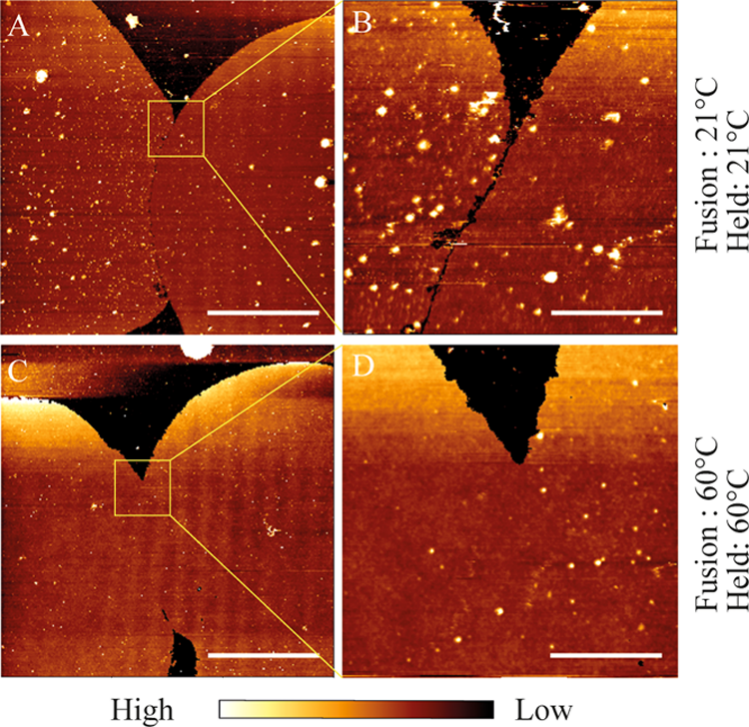
AFM height maps of patches fused and incubated at room temperature (A,B) and at 60 °C (C,D). Scale bars are 10 μm for the main images (A,C), and 1.65 μm for the zoomed in regions (B,D). The color scale shows a height variation of 37 nm (A), 16 nm (B), 13 nm (C), and 14 nm (D).

Adjacent patches fused and kept at room temperature appeared in contact under epifluorescence, but AFM showed a continuous gap of around 100 nm (Figure 5A,B). Polymer patches formed and incubated at 60 °C for 2 h did not show such gaps (Figure 5C,D), explaining the FRAP results in Figure 4. Heating merged the patches, allowing them to equilibrate their fluorescence intensities and to behave as one continuous membrane.

### Formation of Continuous Bilayers

Continuous supported bilayers are desirable for practical applications. An easy method for forming continuous bilayers made of lipids is through the fusion of small unilamellar vesicles (SUVs).^50^ However, block-copolymer SUVs did not fuse to either glass or PDMS substrates (Figure S6) even after incubation at 60 °C: fluorescent images appeared granular under high magnification (Figure S6) and fluorescence did not recover after photobleaching. A similar result was reported by Paxton et al. for PEO22-*b*-PBD37 SUVs.^24^

**Figure 6:**
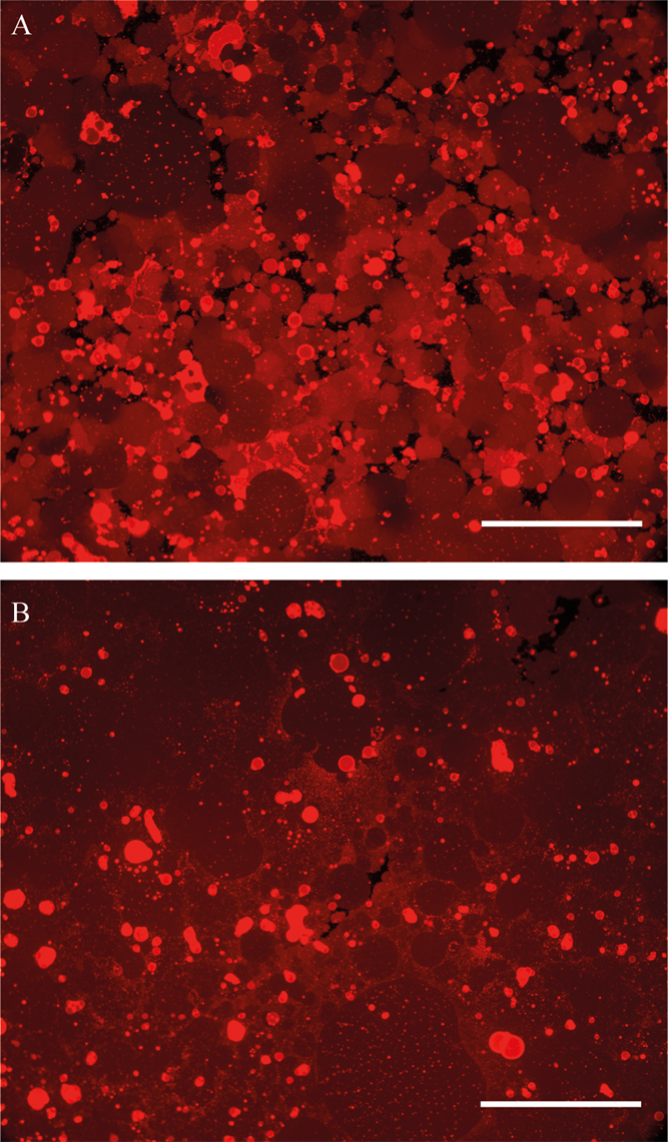
Polymeric membrane before (A) and after (B) annealing at 60 °C on PDMS substrates. (A) After GUV deposition, the membrane created a tapestry of reds which, due to patch boundaries, did not equilibrate. (B) After annealing at 60 °C for 2 h, the patch boundaries merge and a continuous membrane is created. Scale bars show 200 μm.

Since PGUVs readily fused to hydrophilic substrates (Figures 1 and 3), we explored whether the fusion of PGUVs and annealing of the resulting patch edges at 60 °C for 2 h would lead to the formation of continuous polymeric bilayers. The adjacent patches joined at high temperatures into large areas (hundreds of micrometers across) of continuous bilayer cover (Figure 6). However, this method did not produce defect-free bilayers. The large size of the vesicles inhibited homogeneous coverage of the substrate and left gaps in the membrane (Figure 6B). Moreover, GUVs that fused in the gaps between existing patches did not have enough space to unfold on the substrate and contributed a lot of excess membrane area, in the form of protrusions or multiple bilayers. The excess material, which appears bright on the fluorescent images (Figure 6B), could not be easily removed by rinsing. We tried an alternative method where we fused PGUVs at lower concentrations and tried to backfill the gaps with SUVs. However, the SUVs did not fuse, even though previous studies have reported that free membrane edges catalyze the fusion of lipid SUVs.^50^ Additionally, we tried forming bilayers from mixed polymer-surfactant micelles, but the membranes we obtained were not fluid (Figure S7).

### Discussion

PEO14-PBD22 block copolymer can, like phospholipids, be assembled into giant vesicles using electroformation or gentle hydration. However, care must be taken when choosing the fluorescent probe, as we found that the common lipid fluorophores, Rh-DPPE or DilD, did not distribute evenly between PGUVs (Figures 3 and S4), possibly due to the slow diffusion of the probes or their limited solubility in the casted polymer films from which the vesicles are formed.

Like lipid GUVs, PGUVs were able to fuse to hydrophilic glass and PDMS substrates and form patches with distinct heart or circular shapes (Figure 1). The effective vesicle–substrate interaction, which is the sum of the van der Waals attraction, the hydration repulsion, and the entropic repulsion arising from thermal membrane undulations, is sufficient to increase the membrane tension above a critical value leading to vesicle rupture and fusion.^22,23,41,43,51^ However, polymer vesicles needed some additional adjustments to fuse to glass and PDMS. These included (1) baking the plasma-oxidized glass at 200 °C to reduce the hydration repulsion force^44^ or (2) subjecting the vesicles to hypo-osmotic shock, which swells them and increases their membrane tension, as well as reducing the undulation repulsion with the substrate.^51^ However, even with these adjustments we were unable to fuse polymer SUVs to glass and PDMS substrates.

Our experiments show that PGUVs fuse onto both PDMS and glass substrates via a single-burst mechanism. Lipid GUVs on the other hand can fuse in a cascade fashion due to the competition between membrane spreading and fusion pore closure.^43^ The lack of pore closure in PGUVs is consistent with the larger viscosity of polymer membranes^52^ and with their increased edge stability (Figures 4 and 5).

We used the supported membrane patches to characterize the fluidity of the polymeric membranes and the properties of the membrane edges. Supported PEO14-PBD22 membranes appeared fluid, consistent with the ability of fluorophore molecules to diffuse within them (Figure 2). This observation contrasted with previous studies with polymer membranes obtained by micelle^15^ and SUV deposition.^12,24^ The cause of the difference is not entirely clear, but it points to the importance of the bilayer and substrate preparation procedure and/or the choice of fluorescent probe used to measure the diffusion coefficient. None of the fluorophores in this work matched the ones in previous studies. However, we were able to detect slow fluorescence recovery with three fluorophores (Rh-DPPE, DilD, and naphthopyrene) of different molecular structures and sizes (Figure S5).

Supported polymeric membranes were 7 and 8 times (for glass and PDMS substrates, respectively) less fluid than their DOPC analogues (Figure 2a). This difference can be attributed to the larger size of the PEO14-PBD22 polymers (more than twice the *M*_w_ of DOPC) that contributed to higher membrane viscosity.^52^ Therefore, even in PGUVs, undisturbed by the presence of a substrate, the diffusion coefficient of the fluorophores was smaller than in supported lipid membranes. Both supported and unsupported polymeric membranes displayed a significant fraction of immobile fluorophores after photobleaching (Figure 2b). This revealed that not only substrate pinning effects, as in supported lipid bilayers,^53^ but also membrane heterogeneities arising from, e.g., polymer polydispersity, are responsible for the formation of immobile molecular clusters that hinder the diffusion of the fluorophores.

One of the surprising observations in this work was the inability of adjacent polymer patches to merge into a continuous supported bilayer at room temperature. Adjacent patches displayed different fluorescent intensities (Figure 3), and AFM images showed a stable submicron gap between the patch edges, which prevented the equilibration of the fluorophore. The gap disappeared only after incubating the patches at 60 °C for an extended period of time (2 h in our experiments) (Figure 5). Thermodynamically, adjacent membrane patches are expected to merge spontaneously into larger patch areas because this would reduce the length of the energetically unfavorable membrane edge and would increase the energetic gain from adhesion.^50^ The fact that polymer patches remain separate at lower temperatures suggests that their merging is kinetically prevented by repulsion forces, possibly arising from the hydration of the large hydrophilic PEO groups of the polymers. Upon heating, the PEO headgroups are partially dehydrated, which reduces the magnitude and range of repulsive forces between them^54^ and allows them to join. It is also possible that the hydration of the PEO groups leads to spontaneous curvature that stabilizes the edges at room temperature but not at higher temperatures. The stability of the polymer membrane edge agrees well with the single-burst fusion of PGUVs and with the reduced ability of the fusion pores to close and compete with the fusion process.

## Conclusions

In conclusion, we show that supported polymer membranes, tens to hundreds of microns wide, can be easily obtained by giant vesicle fusion, without the need for polymer modifications or substrate functionalization^11,13,14,16−18^ nor the addition of lipids.^12,24^ The supported polymer membranes exhibit some fluidity contrary to previous observations.^12,15,24^ Adjacent polymer patches could be merged by heating to 60 °C. This fusion-annealing protocol can be used easily to create continuous supported polymeric membranes with the caveat that these membranes contain holes and excess material (Figure 6B), which may affect the performance of polymeric bilayers depending on the nature of the application. Importantly, our study characterizes polymer membranes on PDMS substrates, which, as will be shown in subsequent studies, allows us to reveal novel aspects of the mechanical behavior of these systems.

## Supporting Information Available

S2: a comparison of lipid and polymer membrane patches; S3: photobleaching of lipid and polymer membranes; S4: comparative fluorescence and atomic force microscopy images of polymer membranes on glass and PDMS substrates; S5: fluorescence of polymer vesicles prior to fusion; S6: mobility of Rh-DPPE, naphthopyrene, and DilD in polymer membranes; S7: formation of continuous bilayer using small vesicles; S8: mixed surfactant-polymer micelles (9:1); and S9: Supporting references (PDF)
S1: Supporting videos; epifluorescence movie of PGUVs fusing on plasma-oxidized PDMS substrates in 150 mOsmol buffer; frame interval is 0.3 s. A scale bar is 100 μm (AVI)
Epifluorescence movie of PGUVs fusing on plasma-oxidized and baked glass substrates in 150 mOsmol buffer; frame interval is 0.3 s. A scale bar is 100 μm (AVI)

## Supplementary Material

la2c00266_si_001.pdf

la2c00266_si_002.avi

la2c00266_si_003.avi
