# Supplementary material for: Comparative Study of Lipid- and Polymer-Supported Membranes Obtained by Vesicle Fusion
Source: Langmuir. 2022 Apr 26;38(18):5674–81. doi: 10.1021/acs.langmuir.2c00266 (PMC9097520; doi:10.1021/acs.langmuir.2c00266)
Supplement: Supplementary file 1 — la2c00266_si_001.pdf [file la2c00266_si_001.pdf]

Supplementary Information

Comparative Study of Lipid and Polymer Supported Membranes Obtained by Vesicle Fusion

Rachel J. Goodband[1], Colin D. Bain[2], Margarita Staykova[1]**

1. Department of Physics, Durham University, Durham DH1 3LE, UK
2. Department of Chemistry, Durham University, Durham DH1 3LE, UK

Contents

S1: Supplementary Videos S2

S2: A Comparison of Lipid and Polymer Membrane Patches S2

S3: Photobleaching of Lipid and Polymer Membranes S3

S4: Comparative Fluorescence and AFM Images of Polymer Membranes on Glass and PDMS Substrates S4

S5: Fluorescence of Polymer Vesicles Prior to Fusion S5

S6: Mobility of Rh-DPPE, Naphthopyrene and DilD in Polymer Membranes S6

S7: Formation of Continuous Bilayer Using Small Vesicles S6

S8: Mixed Surfactant-Polymer Micelles (9:1) S8

S9: Supplementary References S9

S1: Supplementary Videos

Video S1: Epifluorescence movie of PGUVs fusing on plasma oxidized PDMS substrates in 150 mOsmol buffer, frame interval is 0.3s. Scale bar is 100 µm.

Video S2: Epifluorescence movie of PGUVs fusing on plasma oxidized and baked glass substrates in 150 mOsmol buffer, frame interval is 0.3s. Scale bar is 100 µm.

S2: A Comparison of Lipid and Polymer Membrane Patches

Polymer patches and lipid patches can be formed via the fusion of GUVs onto hydrophilic substrates, Figure S1. Lipid GUVs have been shown to form patches from single fusion events and from cascade (multiple) fusion events from daughter vesicles [S1, S2, S3], which results in irregular patch shapes. Polymer GUVs broadly appear to follow the same fusion dynamics as LGUVs, however, we did not observe cascade fusions with polymer vesicles, Video S1 and S2.

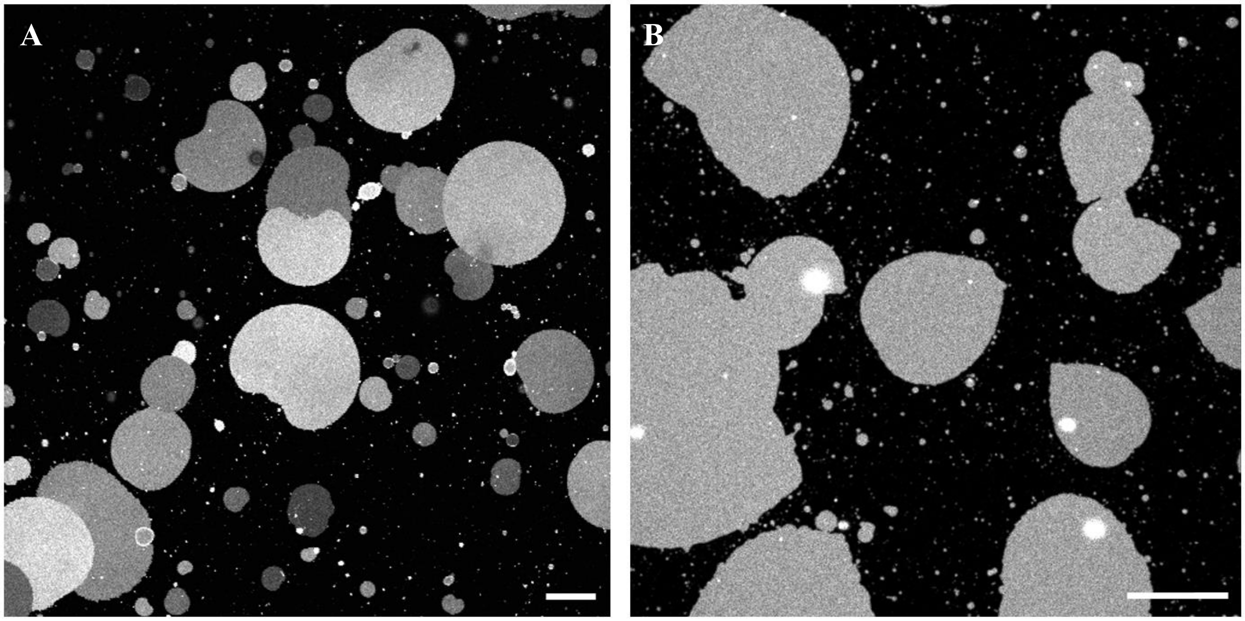

Figure S1: A) Polymer patches and B) lipid patches formed by GUV fusion onto plasma treated PDMS substrates in 150 mOsmol buffer.

S3: Photobleaching of Lipid and Polymer Membranes

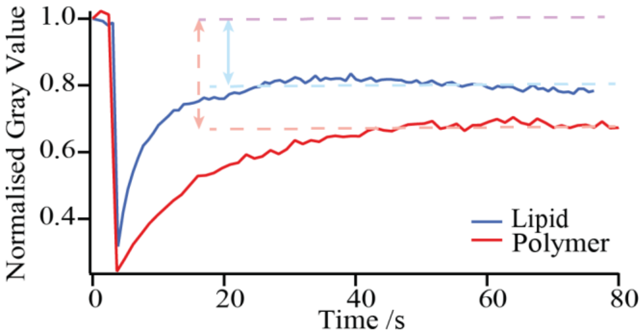

Figure S2: FRAP recovery of bilayer membranes on a PDMS substrate in a hyper-osmotic buffer solution. Blue and red curves show the recovery profiles of a DOPC and PEO14-PBD22 bilayers, respectively. Dashed lines are eye-guides for the plateau of the recovery curve. Light red and blue arrows represent the immobile fraction of the sample as found by the difference between the initial fluorescent value and the plateau of the recovery curve. The grey scale is normalized to the fluorescence of a nearby, non-photobleached membrane patch.

S4: Comparative Fluorescence and AFM Images of Polymer Membranes on Glass and PDMS Substrates

Polymer patches of varying fluorescent intensities did not show any major topological differences in the boundary region or any notable height distinctions (Figure S3). There was however a gap between adjacent patches, observed both on PDMS and glass substrates, which was detected as a height dip. The bilayer heights were found to be around 10 nm throughout the images, indicating that fluorescence intensity had no correlation with the membrane height, i.e. it is not related to multilamellar bilayer structures. PDMS images showed wrinkles due to the plasma treatment of the substrate.

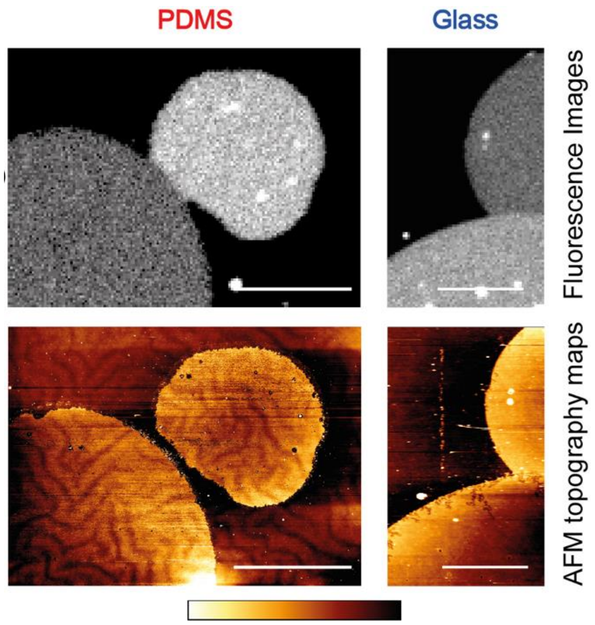

Figure S3: Fluorescence images (top line) and AFM height maps (bottom line) of polymer patches on glass and PDMS substrates. Scale bars show 10 µm in all images and height scales on AFM images show 23 nm and 17 nm for PDMS and glass, respectively.

S5: Fluorescence of Polymer Vesicles Prior to Fusion

Giant polymer vesicles exhibited fluorescence differences after electroformation (Figure S4A) and gentle hydration on cellulose paper (Figure S4B), indicating that the uneven distribution of fluorophores arises in the cast polymer film prior to rehydration.

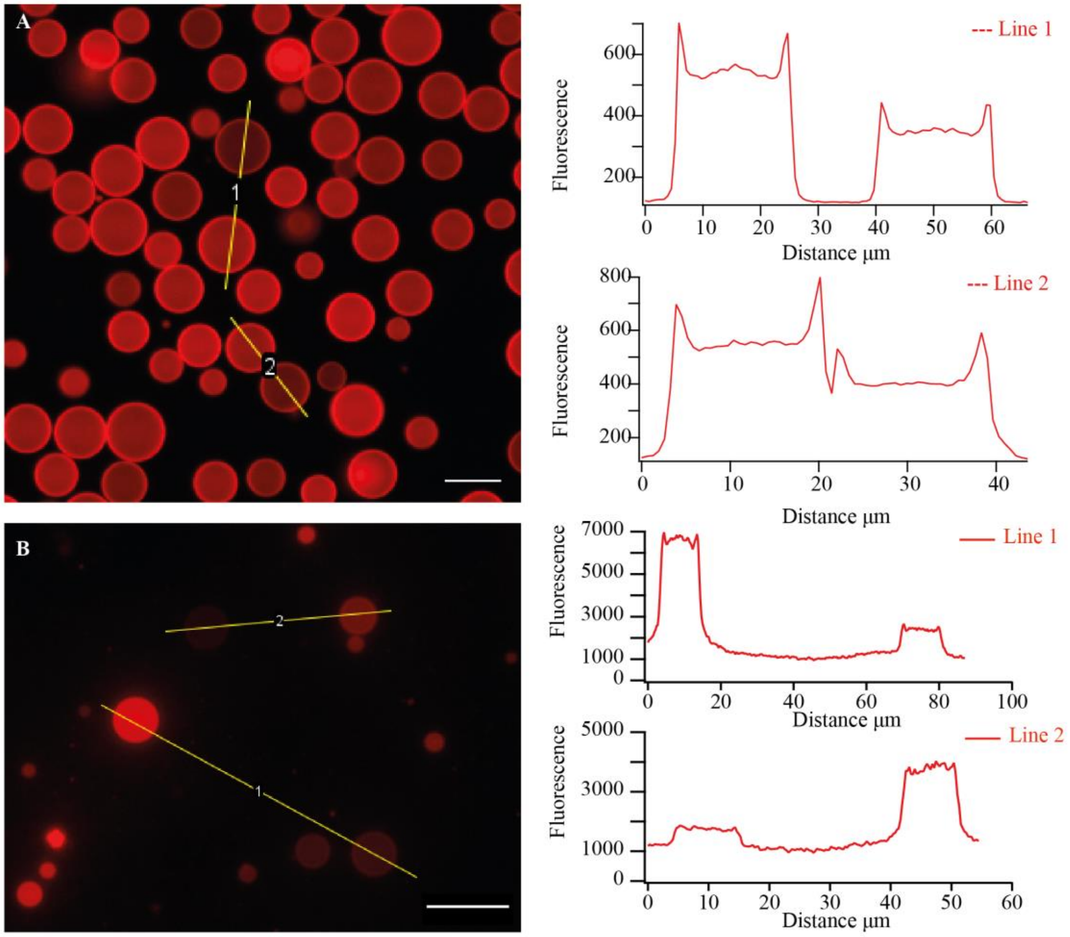

Figure S4: A) Confocal image of PGUVs labeled with Rh-DPPE showing differences in their fluorescence intensity as demonstrated by the two fluorescent line profiles plots. Scale bar is 20 µm. B) Epifluorescence image of GUVs formed by gentle hydration on cellulose paper with fluorescence intensities demonstrated by two fluorescent line profile plots. Scale bar is 20 µm.

S6: Mobility of Rh-DPPE, Naphthopyrene and DilD in Polymer Membranes

All fluorophores (Rh-DPPE, Naphthopyrene and DilD) showed fluorescence recovery after photobleaching (Figure S5). The spread on the naphthopyrene data was large in comparison to the other fluorophores and may have resulted from the relatively low fluorescence signal and faster photobleaching. T-tests assuming unequal variance show that DilD is statistically different from both Naphthopyrene and Rh-DPPE with values of $p = 0.0025$ and $p = 0.00065$ respectively. To a 5% significance, Naphthopyrene and Rh-DPPE are not significantly different with $p = 0.053$.

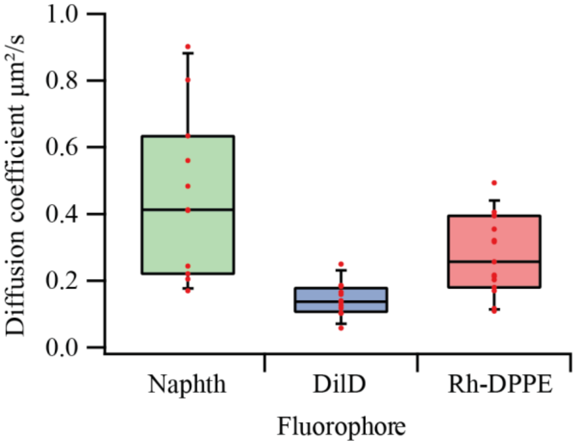

Figure S5: Diffusion coefficients of Naphthopyrene, DilD and Rh-DPPE in polymer bilayers supported on PDMS substrates.

S7: Formation of Continuous Bilayer Using Small Vesicles

FRAP on SUV layers showed no fluorescent recovery (Figure S6). The observed granular fluorescence on samples may be ascribed to unfused SUVs physically adsorbed to the substrate. Even at temperatures of 80 °C, SUVs did not fuse into continuous fluid bilayers.

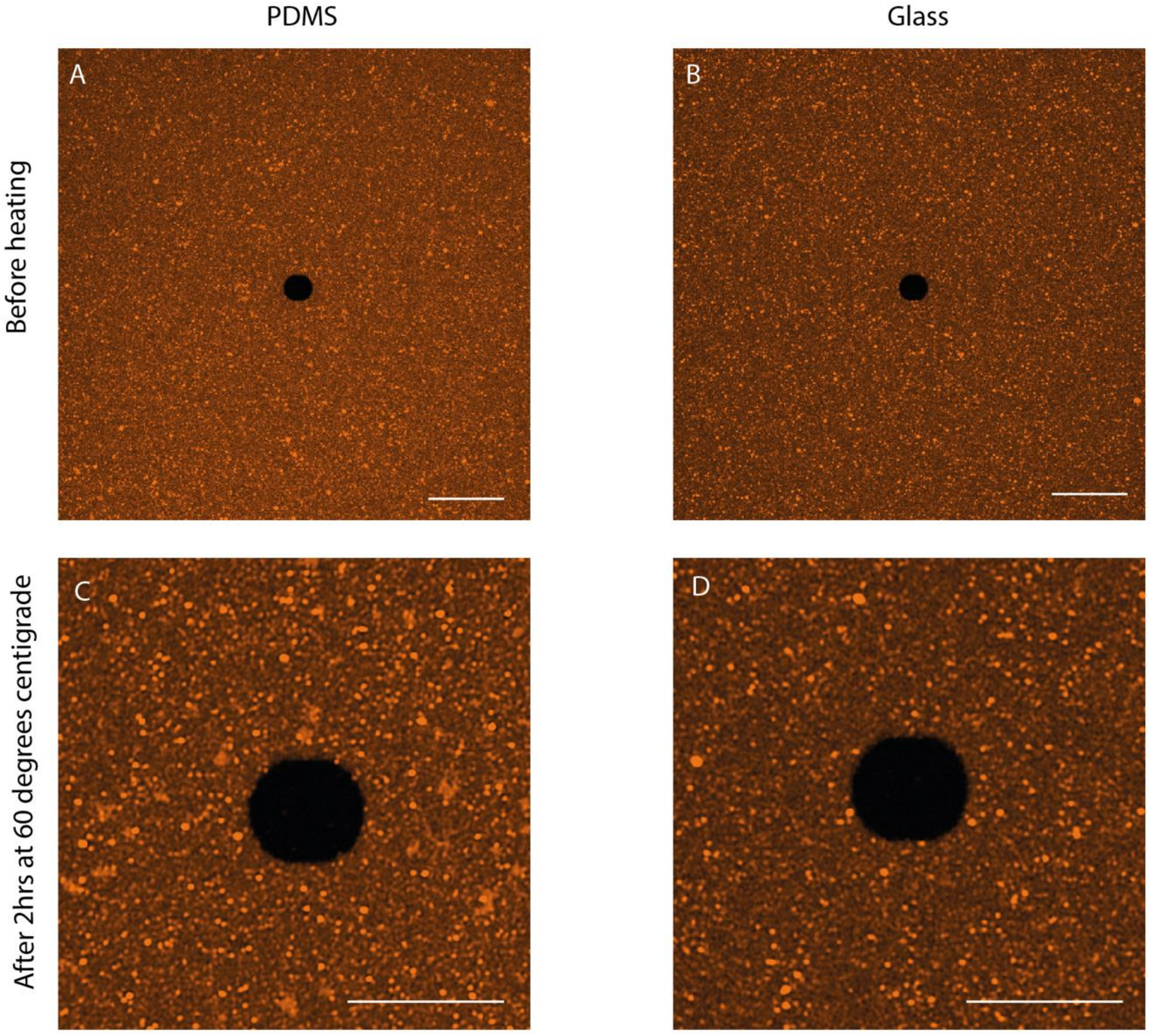

Figure S6: Deposition of SUVs on (A) PDMS and (B) glass substrates result in the formation of granular layers, which do not recover after photobleaching. Same result was obtained after annealing at 60 °C for 2 hrs (C, D). Scale bars A,B - 20 μm, and for C,D - 10 μm.

S8: Mixed Surfactant-Polymer Micelles (9:1)

Following Tiberg et al. [S4], mixed surfactant-polymer micelles were created by the following method. N-Dodecyl-β-maltoside was suspended in chloroform at 13 mg/ml and bath sonicated until the solution was well homogenised (10 mins). Polymer solution with a fluorescent probe (Rh-DPPE) was suspended in chloroform at 4 mg/ml + 1 mol% probe. The surfactant-polymer mixture was used at a 9:1 ratio and mixed well before drying fully with nitrogen and dessicating overnight to remove residual chloroform. The dry surfactant-polymer layer was rehydrated to 0.18 mg/ml with milliQ water, heated to 50 °C and left gently stirring overnight on a hotplate. Glass slides were treated for 5 min. with an air plasma and incubated with micelle solution (0.118 mg/ml) at 60 °C for 30 minutes, before rinsing and leaving for 2 hrs. This process was repeated until a visible fluorescent layer could be seen on the slide (×5). Once fluorescence was apparent, the solution concentration was dropped to below the cmc and the samples were incubated in 0.039 mg/ml solution an additional 2 times to backfill any gaps.

FRAP on bilayers created from micelles showed no fluorescence recovery even after multiple incubations with micelles solution and annealing at 60 °C (Figure S7).

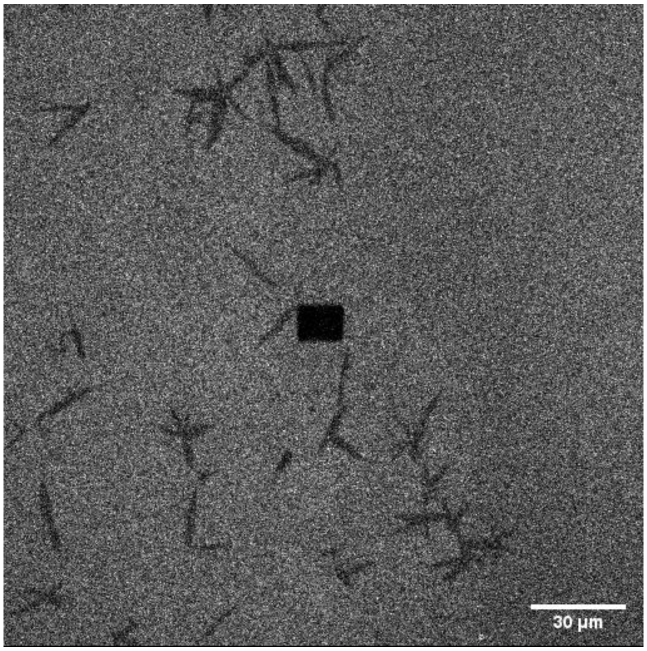

Figure S7: Surface layers formed on glass from mixed surfactant-polymer micelles showing rod-like defects and no recovery after photobleaching. Scale bar shows 30 µm.

S9: Supplementary References

[S1] Hamai, C.; Cremer, P.S.; Musser, S. M. Single Giant Vesicle Rupture Events Reveal Multiple Mechanisms of Glass-Supported Bilayer Formation, *Biophysical Journal*, **2007**, 92, 1988-1999, DOI: 10.1529/biophysj.106.093831

[S2] Kataoka-Hamai, C.; Yamazaki, T. Induced Rupture of Vesicles Adsorbed on Glass by Pore Formation at the Surface–Bilayer Interface, *Langmuir,* **2015**, 31, 4, 1312–1319, DOI: 10.1021/la5042822

[S3] Ngassam, V. N.; Su, W. C.; Gettle, D. L.; Deng, Y.; Yang, D.; Wang-Tomic, N.; Sharma, V. P.; Purushothaman, S.; Parikh, A. N. Recurrent dynamics of rupture transitions of giant lipid

vesicles at solid surfaces, *Biophysical Journal,* **2021***,* 120, 586-597, DOI: 10.1016/j.bpj.2021.01.006

[S4] Tiberg, F.; Harwigsson, I.; Malmsten, M. Formation of model lipid bilayers at the silica-water interface by co-adsorption with non-ionic dodecylmaltoside surfactant. *Eur Biophys J.* **2000,** 29, 196-203, DOI: 10.1007/pl00006646

-----------------------------------end of supplementary information-------------------------------------
